# The Impact of the Dietary Inflammatory Index, Fasting Blood Glucose, and Smoking Status on the Incidence and Survival of Pancreatic Cancer: A Retrospective Case–Control Study and a Prospective Study

**DOI:** 10.3390/nu16223941

**Published:** 2024-11-19

**Authors:** Ga Hyun Lee, Yeon Hee Kim, Sang Myung Woo, Woo Jin Lee, Sung-Sik Han, Sang-Jae Park, Sherry Price, Penias Tembo, James R. Hébert, Mi Kyung Kim

**Affiliations:** 1 Cancer Epidemiology Branch, Division of Cancer Epidemiology and Prevention, National Cancer Center, Ilsandong-gu, Goyang-si 10408, Republic of Korea; ghlee@ncc.re.kr (G.H.L.); yh0227@ncc.re.kr (Y.H.K.); 2 Center for Liver and Pancreatobiliary Cancer, National Cancer Center, Ilsandong-gu, Goyang-si 10408, Republic of Korea; wsm@ncc.re.kr (S.M.W.); lwj@ncc.re.kr (W.J.L.); sshan@ncc.re.kr (S.-S.H.); spark@ncc.re.kr (S.-J.P.); 3 Department of Epidemiology and Biostatistics and Cancer Prevention and Control Program, University of South Carolina, Columbia, SC 29208, USA; sprice@chi-llc.net (S.P.); PTEMBO@email.sc.edu (P.T.); JHEBERT@mailbox.sc.edu (J.R.H.); 4 Department of Nutrition, Connecting Health Innovations LLC, Columbia, SC 29201, USA

**Keywords:** pancreatic cancer, dietary inflammatory index, fasting blood glucose, smoking

## Abstract

Background: Pancreatic cancer (PC), a highly malignant cancer with a poor diagnosis, may be influenced by diet-related inflammation. This study examined the association between dietary inflammatory index (DII) scores and the incidence and prognosis of PC in Korea. Methods: A total of 55 patients with PC were matched with 280 healthy controls (HCs) by age and sex. We also analyzed the combined effects of DII scores and fasting blood glucose (FBG) levels or smoking status on the risk of PC and performed a survival analysis using the Cox proportional hazards method. Results: The DII scores were higher in the patients with PC than those in HCs (odds ratio [OR] = 3.36, confidence interval [CI] = 1.16–9.73, *p* = 0.03), and the effect was larger in women (OR = 6.13, CI = 1.11–33.82, *p* = 0.04). A high DII score was jointly associated with FBG ≥ 126 mg/dL in raising PC risk [OR = 32.5, relative excess risk due to interaction/synergy (RERI/S) index = 24.2/4.34, *p*-interaction = 0.04], indicating a multiplicative interaction. A high DII score combined with ex/current smoker status increased PC risk through an additive interaction (RERI/S = 1.01/1.54, *p*-interaction = 0.76). However, DII scores did not influence disease-free survival. Conclusions: The consumption of an anti-inflammatory diet, coupled with maintaining normal FBG levels and abstaining from smoking, may help reduce the risk of PC by mitigating pancreatic inflammation.

## 1. Introduction

Globally, the incidence and mortality rates of pancreatic cancer (PC) have been increasing continuously [1]. The International Agency for Research on Cancer estimated that PC ranked sixth as the cause of deaths attributable to cancer worldwide in 2022, with 510,600 new cases and 467,000 deaths [2]. The public health burden of cancer has increased with an aging population in South Korea [3]. PC, along with lung and colorectal cancers, is expected to be a major cause of cancer-related mortality in Korea. In line with this trend, the National Cancer Center (NCC) has projected 10,158 new PC cases and 7861 PC-related deaths in Korea by 2024 [4]. This high mortality rate is primarily caused by pancreatic ductal adenocarcinoma (PDAC), which accounts for the majority (90%) of malignant pancreatic tumors [5], being diagnosed at advanced stages with systemic metastases (>50%) [6].

Currently, our understanding of pancreatic tumorigenesis has improved, and studies have confirmed that persistent inflammation significantly contributes to the development and progression of PDAC [7]. Studies that have assessed PC risk factors have revealed that inflammation is linked to several clinical factors, including diabetes mellitus, chronic pancreatitis, cigarette smoking, heavy alcohol consumption, and obesity [8,9,10,11,12,13]. Notably, PC and diabetes have interaction, in which insulin resistance caused by diabetes results in hyperinsulinemia and hyperglycemia, and the combined effects of inflammation and obesity raise the risk of PC [14]. Diet is also considered to be a modulator of systemic inflammation. A variety of dietary components directly influence cytokine signaling and the expression of inflammatory markers [15,16]. Dietary constituents common in a Western diet (e.g., saturated fat and simple carbohydrates) are pro-inflammatory, while those eaten in healthy diets in East and South Asia are anti-inflammatory [17,18]. The dietary inflammatory index (DII) was developed to comprehensively assess the inflammatory potential of 45 food parameters in dietary intake by evaluating the expression of six inflammatory biomarkers: interleukin-1 beta (IL-1β), IL-4, IL-6, IL-10, C-reactive protein (CRP), and tumor necrosis factor-alpha (TNF-α) [19].

Research on the association between the DII score and PC risk has found inconsistent results [20,21,22,23,24,25,26], with several studies showing an increased risk [21,22,24]. Furthermore, although prospective studies have investigated the relationship between PC risk and DII [25], there is a lack of studies examining their impact on the prognosis. In contrast, high DII scores have been reported to increase not only the risk of cancer incidence but also mortality associated with other cancers, including breast, colorectal, liver, and prostate cancers [27,28,29,30,31,32,33,34]. A comprehensive understanding of PC risk factors related to diet and inflammation remains unclear.

The purpose of this retrospective case–control and a prospective study was as follows: (1) to examine the association between the DII score and the risk of PC; (2) to investigate the interaction of the DII score with glycemic status or smoking. Additionally, we aimed to assess the impact of the DII score, fasting blood glucose (FBG) levels, and smoking status on the PC-related recurrence and death attributable to PC.

## 2. Materials and Methods

### 2.1. Study Population

**Figure 1:**
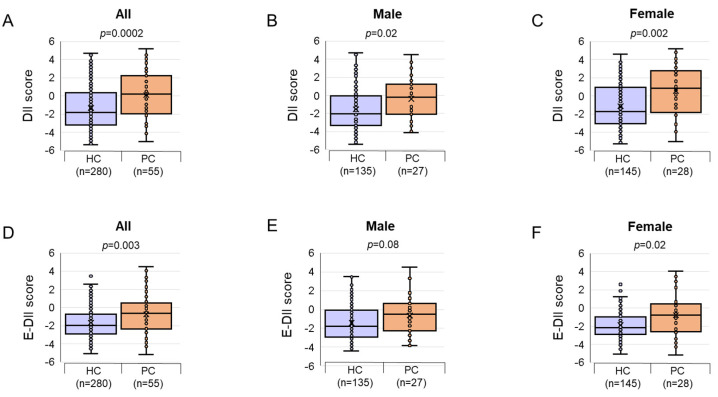
The comparison of the DII and E-DII score for 55 PC cases and 280 HCs, stratified by sex. (**A**) DII scores between the groups; (**B**) DII scores between the groups for males; (**C**) DII scores between the groups for females; (**D**) E-DII scores between the groups; (**E**) E-DII scores between the groups for males; (**F**) E-DII scores between the groups for females. The data are presented as boxplots. *p*-values were calculated using the Mann–Whitney *U* test for DII and E-DII scores across all three groups. Abbreviations: DII, dietary inflammatory index; E-DII, energy-adjusted dietary inflammatory index; PC, pancreatic cancer; HC, healthy control.

This hospital-based study was conducted from 2019 to 2023 at the National Cancer Center (NCC) in Korea. It was designed in two studies: a retrospective case–control study including subjects with PC and healthy controls (HCs); and a prospective study including only subjects with PC. Eligible patients who underwent medical health checkups and cancer screening were those aged 19–74 years with newly diagnosed PC. Control group consisted of healthy individuals with neither a history of cancer nor a genetic predisposition for cancer, or any family history of cancer, who were recruited from the same hospital from which the patients were enrolled. The HCs were matched to the patients at a 1:5 ratio based on age and sex (Figure 1). All participants provided written informed consent and both groups agreed to participate in the study. This study was performed in accordance with the protocols approved by the Institutional Review Board (IRB) of the NCC (IRB no. NCC 2019-0116, approval date: 3 June 2019), and it adhered to the Declaration of Helsinki.

### 2.2. Data Collection

Data were collected using a structured questionnaire to acquire information on sociodemographic covariates (age, sex, and educational level), smoking status, and alcohol drinking. There were three categories for educational level: elementary school or lower, middle school to high school, and college or higher. ‘Current’ smokers were considered individuals who had smoked more than 400 cigarettes throughout their lifetime and continued to smoke at the time of the interview. ‘Ex-smokers’ were those who had smoked over 400 cigarettes in a lifetime but had ceased smoking. ‘Never’ smokers were defined as having smoked fewer than 400 cigarettes or never smoked at all. Alcohol drinking experience was also divided into three categories, ‘Current’ (present drinking), ‘Ex’ (alcohol consumption quitters), and ‘Never’ (those with no history of alcohol drinking). Anthropometric measurements were performed by calculating the body mass index (BMI), defined as weight (kg) divided by height squared (m^2^), as an index of obesity. Results of the FBG test and personal medical histories (including the date of the diagnosis and cancer stage) were acquired from all participants at the NCC. The glycemic indicators of the participants were categorized as FBG <126 mg/dL (non-diabetes) and ≥126 mg/dL (diabetes) in accordance with the Clinical Practice Guidelines for Diabetes of the Korean Diabetes Association [35]. Radiological imaging was typically performed using computed tomography to determine PC staging. The findings were reviewed according to the American Joint Committee on Cancer (AJCC) staging manual [36]. Death records were obtained from an electronic medical record database.

### 2.3. Dietary Assessment and DII Score Calculation

A detailed description of the DII is provided elsewhere [19]. All participants completed a standardized food frequency questionnaire (FFQ) that included 95 food items to assess their average daily food intake over the past 1-year period. To classify the frequency of food intake, each food item was categorized into the following nine query items: rarely, once monthly, two–three times monthly, once or twice weekly, three–four times weekly, five–six times weekly, once daily, twice daily, and more than three times daily. The portion sizes were assumed to be one-half serving, a standard serving, and one and a half times the standard serving for all items, and the FFQs were reviewed by a trained interviewer. Total calorie and nutrient intakes were computed based on the FFQ-derived dietary data using the Computer Aided Nutritional Analysis Program (CAN-Pro) 5.0 and 6.0, the nutritional assessment software developed by the Korean Nutrition Society. Participants with implausible energy intake (males <800 kcal or >5000 kcal, females <500 kcal or >4200 kcal) were excluded from the analyses. Each nutrient item in the CAN-Pro was matched to the 45 food parameters that comprised the DII score, including macronutrients, micronutrients, and bioactive ingredients. To quantify the dietary inflammatory potential, the DII^®^ scores were calculated for 45 food parameters based on an extensive review of over 6000 articles published from 1950 to 2010 and classified into pro-inflammatory and anti-inflammatory diets (listed in Sreeja et al.) [37,38]. The energy-adjusted DII (E-DII^TM^) allows for energy adjustment by calculating the DII per 1000 kilocalories consumed [37]. In this study, 30 food parameters were available to calculate the DII score of diets: energy, carbohydrates, total fat, protein, fiber, vitamin A, β-carotene, vitamin C, vitamin D, vitamin E, thiamin, riboflavin, niacin, vitamin B6, folic acid, vitamin B12, magnesium, iron, zinc, selenium, cholesterol, saturated fatty acids (SFAs), monounsaturated fatty acids (MUFAs), polyunsaturated fatty acids (PUFAs), *n*-3 PUFA, *n*-6 PUFA, garlic, green/black tea, caffeine, and alcohol. To calculate the DII, the amount reported on the FFQ was subtracted from the standard global mean, and the result was then divided by the global standard deviation to compute a z-score. All z-scores were transformed into percentiles to minimize the effect of right skewing, which were then multiplied by the inflammatory effect score of each food parameter according to Shivappa et al. [19]. The overall DII score for each participant was calculated by summing the resulting values. A DII score above zero indicated a pro-inflammatory diet, while a score below zero represented an anti-inflammatory diet. Higher DII and E-DII scores indicated an increased inflammatory potential of the diet.

### 2.4. Statistical Analysis

To describe continuous variables, the median and interquartile ranges are used (Q1–Q3), and categorical variables are presented as frequencies (%). Continuous variables, such as age and BMI, were analyzed using the Mann–Whitney *U* test, while categorical variables, including sex, age (<60 years, ≥60 years), education level, BMI (<23.0, 23.0–24.9, ≥25.0), smoking, and alcohol drinking, were analyzed using the chi-square test. After adjusting for the significant covariates, a logistic regression analysis was performed to assess the association of these variables with PC. To focus on the main effects of DII, the logistic regression analysis was conducted on the DII, E-DII, and DII components using the first (or lowest) tertile as the reference category. The multivariate model was adjusted for the variables including sex, age, education, BMI, and smoking status. The *p*-values for trends were calculated by assessing the continuous scale and assigning the median values to each quantile, treating the values as continuous variables. To determine the combined effect of the DII score and FBG level or smoking status, the relative excess risks due to interaction/synergy (RERI/S) were used as indices of the additive interaction for the dichotomized exposures [39,40]. A synergistic effect between two risk factors was indicated by RERI > 0 and S > 1. The *p*-values for interactions represented the multiplicative interaction of the binary factors (DII score × FBG level; DII score × smoking status). Using the Cox proportional hazards model, the prognosis of the PC group was examined by assessing disease-free survival (DFS), overall survival (OS), and relapse-free survival (RFS). OS was calculated from the date of the PC diagnosis until the date of the latest follow-up or death from any cause. DFS was defined as the time interval between the date of the diagnosis and cancer recurrence or death due to any cause. RFS was computed as the time from the diagnosis to the date of the last follow-up or recurrence. The follow-up time was represented on the *x*-axis and the DII score on the *y*-axis, with survival or mortality illustrated using Cytoscape (version 3.10.2, https://cytoscape.org/).

## 3. Results

### 3.1. General and Clinical Characteristics of Patients in the PC and HC Groups

The distribution of the 55 patients with PC and 280 HCs according to the sex, age, education level, and the other selected covariates is summarized in Table 1. The PC group was associated more with lower education levels, lower average BMI (kg/m^2^), infrequent alcohol consumption, and higher FBG levels than the HC group. Patients diagnosed with stage 4 disease were significantly overrepresented in the PC group. The variables statistically associated with PC risk included education level above middle school, BMI ≥ 25 kg/m^2^, being a drinker, and FBG ≥ 126 mg/dL (Table 1). The results showed that the higher the educational level and BMI, the lower the risk of PC. There was no association between smoking and the risk of developing PC in this study. Among the groups, current alcohol drinkers had the lowest odds ratio (OR) compared to ex-drinkers and non-drinkers. The variable that showed the highest OR level was FBG ≥ 126 mg/dL.

### 3.2. Association of DII Score with PC Risk

**Figure 2:**
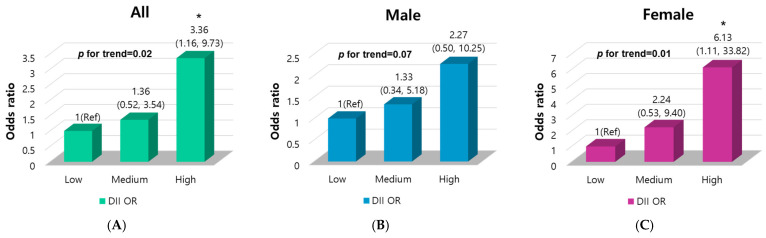
Multivariable logistic regression analyses of the three levels of DII scores for PC risk by sex. DII was adjusted for sex, age, BMI, smoking, and energy intake. DII was divided into three ranges, low, medium, and high DII, from the lowest to the highest. ORs and 95% CIs were evaluated to determine the relationship between the three levels of DII scores and PC risk using a multivariable logistic regression analysis for PCs and HCs. (**A**) ORs and 95% CIs among the three levels of DII; (**B**) ORs and 95% CIs among the three levels of DII for males; (**C**) ORs and 95% CIs among the three levels of DII for females. * *p* < 0.05. The *p*-values for trends were computed by treating the values as continuous variables, evaluating the continuous scale, and allocating median values to each quantile. Abbreviations: OR, odds ratio; CI, confidence interval; Ref, reference value; DII, dietary inflammatory index; PC, pancreatic cancer; HC, healthy control.

Patients with PC had markedly higher DII scores than HCs, which is indicative of a pro-inflammatory diet. Furthermore, similar results were obtained in both males and females (Figure 1). Multivariable logistic regression analyses were used to evaluate whether the DII scores could be risk factors for PC (Figure 2). In the three-dimensional analysis, the DII scores were divided into three ranges using the group with a low DII score as a reference. The OR for the medium and high groups showed a positive association between DII level and PC risk, revealing a 3.36-fold higher risk of PC in the high-DII group compared to the low-DII group. No associations were observed between the highest and lowest tertiles of the E-DII scores (Table S1). Taken together, we found that a higher DII score was significantly related to an increased PC risk. Based on these results, we ascertained that dietary inflammatory potential might contribute to inflammation, increasing the risk of PC. An increased effect of a pro-inflammatory diet on PC risk was observed in women. These findings suggest that women who consumed a pro-inflammatory diet with a high DII score may be more susceptible to PC risk than men.

### 3.3. Associations Between DII Components and PC Risk

The ORs and 95% CIs of PC risk for the selected 30 food parameters by DII tertiles among the 55 PC cases and 280 HCs are shown in Table S2. In the multivariate model, the consumption of DII components, including magnesium, *n*-3 PUFA, and *n*-6 PUFA, was positively associated with PC risk. In contrast, the intake of 17 out of 30 food parameters showed an inverse association with PC risk, which are listed as follows: vitamin A, β-carotene, vitamin D, vitamin E, vitamin C, thiamin, niacin, vitamin B6, folic acid, vitamin B12, iron, cholesterol, and MUFA. We confirmed that magnesium, *n*-3 PUFA, and *n*-6 PUFA increased the risk of PC, although these are known to be anti-inflammatory in nature [38].

### 3.4. Association of FBG Levels with PC Risk

The FBG level was positively associated with PC risk (Table 2). FBG levels were categorized into two groups based on the criteria for diagnosing diabetes to investigate the relationship between each FBG range and PC risk. The FBG level was identified as a strong risk factor for PC, stronger than other biochemical indicators, regardless of sex. Interestingly, women exhibited a considerably higher risk of developing PC than men in all three FBG ranges. This implies that glycemic control is crucial for reducing the risk of PC in women.

### 3.5. Effect of Combination of DII Score with FBG Level or Smoking Status on PC Risk

We further examined the interaction between the DII score and fasting glycemia values or smoking status for PC risk (Table 3). The DII score was divided into two levels—high DII and low DII—to investigate the effect of the combination of the DII score and blood glucose level or smoking status. Interestingly, the observed joint association of FBG levels above 126 mg/dL with high DII scores showed a remarkable 32.5-fold increased risk for PC relative to that of the reference (Table 3).

We confirmed that a high FBG level (≥126 mg/dL) was the best indicator for increased risk of PC among the variables, which showed a noteworthy synergistic effect with a high DII score on a multiplicative-scaled interaction. In this study, although smoking was not associated with PC risk (Table 1), past or current smokers with a DII score above the median had a 3.9-fold higher risk of developing PC than non-smokers with low DII scores (Table 3). The interaction between the two variables was found to have an additive synergistic effect in the RERI/S analysis; however, no multiplicative interaction was observed.

### 3.6. Effect of Variables on DFS and OS

**Figure 3:**
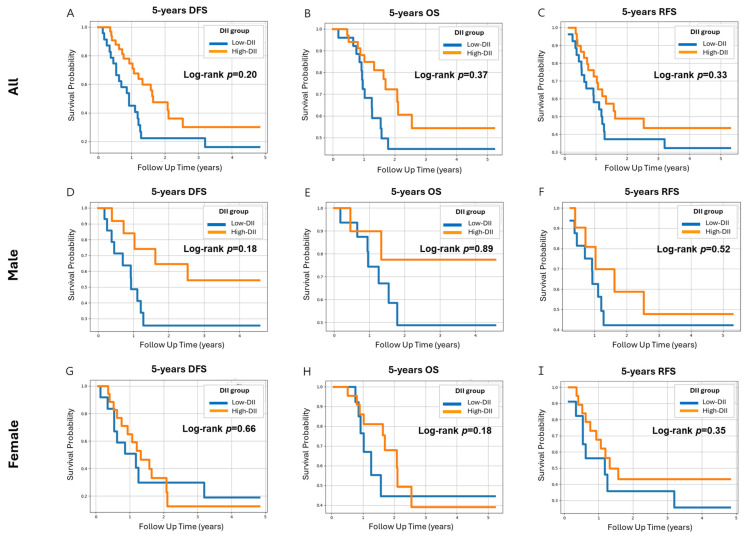
The adjusted Cox proportional hazards model for 5-year DFS, 5-year OS, and 5-year RFS of patients with PC by the DII score. The prognosis of the 55 PC cases was analyzed to assess DFS, OS, and RFS using the Cox proportional hazards model. This model was adjusted for sex, age, BMI, and energy intake. The DII score was divided into two groups: low DII and high DII. Survival curves based on DII scores were analyzed among 55 PCs for (**A**) 5-year DFS, (**B**) 5-year OS, (**C**) 5-year RFS, (**D**) 5-year DFS for males, (**E**) 5-year OS for males, (**F**) 5-year RFS for males, (**G**) 5-year DFS for females, (**H**) 5-year OS for females, and (**I**) 5-year RFS for females. Abbreviations: DFS, disease-free survival; OS, overall survival; RFS, recurrence-free survival; PC, pancreatic cancer; DII, dietary inflammatory index.

The effect of the DII score on death and recurrence of PC was assessed using the Cox proportional hazards model. During the follow-up, 34 PC-related deaths were recorded. No alteration in DII scores was observed in the 5-year DFS, indicating that DII scores were not associated with the prognosis in PC (Figure 3). Similarly, no differences in 5-year outcomes were observed between the groups with high and low FBG levels, suggesting that FBG levels were not linked to the PC prognosis (Figure S1). In contrast, the Cox proportional hazards model for 5-year OS demonstrated that smoking reduced survival probability in male patients with PC over 5 years of follow-up (Figure S2). Male smokers (both ex-smokers and current smokers) with PC showed an increased death rate compared to non-smokers.

## 4. Discussion

We examined the association between the DII scores and PC risk, along with their interactions with FBG levels or smoking status. Our results showed that DII scores were notably higher in patients with PC than in HCs and that the elevated DII scores were related to an increased risk of PC, particularly in women. Additionally, a high DII score combined with FBG levels ≥126 mg/dL was associated with a markedly higher risk of PC, indicating a multiplicative interaction. Similarly, an additive interaction was observed when high DII scores were combined with smoking status. However, no significant association was found between the DII scores and DFS, indicating a lack of influence on the prognosis.

In this study, the significant association between the DII score and PC risk was supported by the results of the multivariable logistic regression analysis, in which patients with PC had higher DII scores than the HCs. The multivariable logistic regression analysis demonstrated that the OR was higher for PC cases with high DII scores. Additionally, compared to the lowest level, the adjusted OR for PC at the highest level of the DII score was 3.36 (*p* = 0.03), indicating a significantly higher risk of PC in individuals with higher DII scores who consumed pro-inflammatory diets [19]. Similar results have been reported in meta-analyses and previous epidemiological studies [20,21,22,23,24,25,26]. One such meta-analysis and systematic review, which included four case–control studies (2737 cases and 4861 controls) and two prospective cohort studies (634,705 participants, 3152 of whom were incident cases), reported that the risk of PC was 45% higher for those with the highest DII score compared to individuals with the lowest DII score (risk ratio = 1.45; CI = 1.11, 1.90; *p* = 0.006) [20]. A closer look at these studies reveals inconsistent findings regarding the association between DII scores and PC risk. Italian case–control studies found that participants who consumed a pro-inflammatory diet were at a higher risk of developing PC [21,24]. Similar outcomes were obtained in a case–control study by Antwi et al. (OR_Q5_ versus _Q1_ = 2.54, CI = 1.87–3.46, *p*-trend < 0.0001) [22] and a multiethnic replication study (OR_Q5_ versus _Q1_ = 2.20, CI = 1.85–2.61, *p*-trend < 0.001) [23]. However, two prospective cohort studies conducted in the USA showed conflicting results [25,26]. Although no conclusion has been reached, our study supports the idea that diets with high inflammatory potential may be potentially associated to PC incidence.

Physiological processes may explain the crucial role of diet in influencing PC risk. Our daily diets are complex mixtures comprising numerous dietary components, including macronutrients, micronutrients, and polyphenols [41]. Over an extended period, food intake exerting pro-inflammatory effects contributes to the production of multiple cytokines and low-molecular-weight proteins that regulate inflammatory responses in both tumors and various non-cancer cells, including adipose tissue [7]. Diet-driven chronic inflammation may be involved in pancreatic tumorigenesis by increasing the levels of cytokines, which stimulate the generation of pro-inflammatory enzymes and reactive oxygen species (ROS) [42]. ROS triggers cellular damage, resulting in DNA damage and subsequent mutations, DNA adduct formation, and the development of pancreatic tumors [43]. Additionally, diet may affect immune cells by interacting with host metabolism and immunity through the intestinal microbiota, generating various metabolites, such as short-chain fatty acids [44]. Furthermore, the chronic consumption of a high-inflammatory diet leads to the release of excessive growth factors, such as platelet-derived growth factor and transforming growth factor-beta, which are known to cause pancreatic fibrogenesis [45].

High FBG levels were associated with the highest OR for PC risk among the variables, especially in women, in this study. Kim et al. also identified that FBG levels within prediabetic ranges were linked to PC incidence in 19,050 Korean participants (HR_Q4_ versus _Q1_ = 2.31, CI = 1.68–3.17, *p*-trend < 0.001) [46]. Furthermore, the observed joint effect of high DII scores and FBG levels resulted in a large (nearly 32.5-fold) increase in PC risk compared to the reference value in our study. A case–control study provided similar results: the risk of PC was more than five times higher in individuals with a DII score above the median and a history of diabetes (OR = 5.80, CI = 4.17–8.07), and six times higher in those who had diabetes for ≥5 years with a DII score above the median (OR = 6.03, CI = 3.41–10.65) [22]. This study also demonstrated that the synergistic effect of high DII scores and FBG levels was stronger than the combined effect with smoking exposure [22]. The underlying mechanism by which diet-related inflammation may synergistically increase the risk of PC in conjunction with high FBG levels is not completely understood; however, some potential explanations may be provided. For example, the consumption of a highly inflammatory diet, combined with hyperglycemia, may stimulate the secretion of cytokines and trigger cancer-related inflammation [47]. Glucose is metabolized to pyruvate via glycolysis, which is a sequence of ten enzyme-catalyzed reactions [48]. Advanced glycation end products (AGEs) are synthesized by combining reduced sugars with the amino groups of nucleic acids, lipids, or proteins via AGE-associated pathways, some of the collateral pathways associated with glycolysis [49]. AGEs interact with receptor advanced glycation end products (RAGEs), which activate various signaling pathways that trigger oxidative stress, cellular DNA damage, carcinogenesis, and inflammation [50]. High glucose conditions accelerate AGE accumulation, and the RAGE/AGE interaction leads to the activation of nuclear factor-kappa beta (NF-κB), production of ROS, and upregulation of inflammatory cytokines (IL-1β, IL-6, TNF-α) in pancreatic beta cells [51,52,53]. Moreover, hyperglycemia induces the activation of transforming growth factor-β1 (TGF-β1), a tumor promoter, which causes pancreatic cell proliferation and apoptosis [54]. TGF-β1 stimulates the phosphorylation of the Smads signaling cascades, acting as intracellular mediators [55]. The stimulated Smad proteins decrease the expression of epithelial cadherin in epithelial cells, including pancreatic ductal cells, and result in epithelial–mesenchymal transition, which is defined as the cellular morphologic transition from an epithelial to a mesenchymal phenotype as a process of tumor progression [56]. Although the DII score and FBG levels can be independent factors contributing to the increase in PC risk, the strong multiplicative effects are observed when they are combined (Figure S3).

We further examined the joint association of the DII score with smoking status for PC risk, which is well-known factors contributing to the fatal outcomes associated with the incidence and progression of PC [57,58]. Numerous studies have reported an association of smoking with an increased risk of PC [59]. However, in our study, smoking exposure was not independently associated with PC risk but showed synergistic effects with high DII scores. Among the 55 PC participants, non-smokers accounted for 26 (47.3%), while current smokers comprised only 6 (10.9%). Therefore, it is estimated that smoking status had no effect on PC incidence in this study due to the limited proportion of smokers. Smoking is a well-established promoter of PC [60]. Carcinogenic compounds, such as cigarette toxins and metabolites produced by burning tobacco, generate ROS that induce lipid peroxidation in epithelial cells and other cell membranes by activating oxidation-sensitive cellular pathways [61]. Activated immune cells promote the secretion of pro-inflammatory factors, which activate NF-κB and activator protein-1, and induce the release of inflammatory cytokines, such as IL-8 and TNF-α [62,63]. These inflammatory mediators enable cell-mediated immunity to accelerate tumor progression [58]. Antwi et al. demonstrated that the combination of current smoking and the DII score above the median was jointly associated with PC risk (OR = 4.79, CI = 3.00–7.65), which was greater than that predicted under additive and multiplicative interactions [22]. Based on these findings, we speculated that an anti-inflammatory diet and smoking cessation would reduce inflammation and therefore reduce PC tumorigenesis.

To better elucidate the effects of the DII score on survival probability, a Cox proportional hazards model analysis was performed comparing the 5-year DFS, 5-year OS, and 5-year RFS. The results of these analyses indicated a lack of association between the DII score and death or survival. Similarly, Nagle et al. reported that a high-DII diet modestly raises the risk of ovarian cancer but is not associated with ovarian cancer-specific survival [64]. Moreover, in the Cox proportional hazards model, no significant association was observed between FBG levels and 5-year DFS or OS. These results are consistent with those of Zhang et al., showing that the FBG levels were not related to the OS of patients with PC [65]. However, smoking decreased the 5-year OS rate in male patients but was not associated with the 5-year DFS and 5-year RFS. In addition, a retrospective study cohort involving 2323 patients with PDAC identified smoking as a statistically significant independent prognostic marker for survival [66].

In this study, the sex-stratified analysis provided a clearer understanding of the impact of sex on the variables affecting PC risk. The DII scores of female patients were higher than those of male patients. Additionally, we observed that the OR for females increased with higher DII levels compared to males. Although the DII score affected PC risk, it was evident that women were more sensitive to dietary inflammatory potential than men. In contrast, men exhibited significantly lower OS related to smoking compared to diet. The present study has several strengths. First, it contributes to the literature by demonstrating that the DII scores are related to death due to PC, especially in combination with other variables, such as FBG levels or smoking status. Second, this is the first retrospective case-control study and a prospective study to analyze the association of the DII scores with PC incidence and death. The results of this study may serve as a reference for large-scale studies of PC related to DII scores. Third, the DII uses a large literature base to quantify dietary exposure for each participant based on its association with six well-known inflammatory biomarkers [38]. Fourth, the sex-stratified analysis allowed us to identify the effects of sex-specific differences in lifestyle on PC risk, which will help in developing preventative approaches.

Despite its strengths, this study includes several limitations. First, the sample size was small, which limited statistical inference. Second, recall bias may occur in case–control studies. It is well known that FFQs are subject to information biases. Another limitation was that 30 of the 45 food parameters were included in calculating the DII score. It would be better to research the underlying mechanisms that connect diet-driven inflammation, metabolic health, and PC progression, as well as investigate the possible effects of specific dietary interventions for patients.

In summary, our study primarily used the DII score as an indicator of PC risk. A pro-inflammatory diet was found to have a significant impact on the incidence of PC, although it seemed to have less impact after PC progression. Elevated FBG levels (≥126 mg/dL) or smoking synergistically increased PC risk when combined with higher DII scores. Notably, we observed a multiplicative interaction between the elevated FBG levels within the diabetic range and the DII scores above the median. Our observations strongly indicate that adopting a diet with a lower inflammatory potential, controlling fasting glycemia, and quitting smoking are effective strategies to mitigate the risk of PC.

## 5. Conclusions

In conclusion, this study demonstrated a significant association between higher DII scores and increased risk of PC, particularly in women. These findings also suggest that elevated FBG levels, when combined with a high DII score, further amplify PC risk, indicating a synergistic effect between metabolic factors and diet-induced inflammation. However, no significant association was found between DII scores and DFS, suggesting that while inflammatory diets may contribute to the onset of PC, they may not influence the prognosis. These results underscore the importance of dietary and metabolic interventions in reducing PC risk and highlight the need for further research to investigate potential preventive strategies focusing on inflammation and glucose regulation.

## Figures and Tables

**Table 1:** General and clinical characteristics of the study participants and results of the univariable logistic regression analysis for PC risk.

Characteristics	PC	HC	*p* ^a^	OR (95% CI) ^b^	*p* ^c^
Sample Size, n (%)	(n = 55)	(n = 280)			
Sex					
Male	27 (49.1%)	134 (47.9%)	1.00	Ref	
Female	28 (50.9%)	146 (52.1%)		1.04 (0.58, 1.85)	0.91
Age, years	63.0 (54.5–67.5)	62.0 (56.0–66.0)	0.74		
<60	23 (41.8%)	125 (44.6%)	1.00	Ref	
≥60	32 (58.2%)	155 (55.4%)		0.97 (0.54, 1.74)	0.92
Education level					
<Middle school	8 (15.1%)	7 (2.50%)	<0.0001	Ref	
Middle–high school	31 (58.5%)	131 (46.8%)		0.21 (0.07, 0.61)	0.005
≥College	14 (26.4%)	142 (50.7%)		0.09 (0.03, 0.27)	<0.0001
BMI, kg/m^2^	22.9 (21.1–24.6)	24.2 (22.3–26.4)	0.0002		
<23.0	28 (50.0%)	90 (32.1%)	0.005	Ref	
23.0–24.9	17 (30.9%)	78 (27.9%)		1.43 (0.73, 2.80)	0.30
≥25.0	10 (18.2%)	112 (40.0%)		0.41 (0.18, 0.94)	0.04
Smoking					
Never	26 (47.3%)	158 (56.4%)	0.39	Ref	
Ex	23 (41.8%)	91 (32.5%)		1.54 (0.83, 2.85)	0.17
Current	6 (10.9%)	31 (11.1%)		1.18 (0.45, 3.10)	0.74
Alcohol drinking					
Never	17 (30.9%)	70 (25.0%)	<0.0001	Ref	
Ex	18 (32.7%)	30 (10.7%)		2.47 (1.12, 5.44)	0.02
Current	20 (36.4%)	180 (64.3%)		0.46 (0.23, 0.92)	0.03
FBG, mg/dL	127.5 (107.3–153.5)	99.0 (92.0–111.0)	<0.0001		
<126	26 (48.1%)	253 (90.7%)	<0.0001	Ref	
≥126	28 (51.9%)	26 (9.32%)		10.1 (5.21, 19.7)	<0.0001
AJCC staging					
Unknown	8 (14.5%)				
1	4 (7.30%)				
2	7 (12.7%)				
3	9 (16.4%)				
4	27 (49.1%)				

Data are presented as the median and interquartile range (Q1–Q3) for continuous variables and n (%) for categorical variables. ^a^ *p*-Values were calculated using the Mann–Whitney *U* test for continuous variables and the chi-square test for categorical variables between PCs and HCs. ^b^ OR and 95% CI for variables were analyzed using univariate logistic regression to evaluate the relationship between PCs and HCs. ^c^ *p*-Values were calculated using a univariate logistic regression analysis to evaluate the relationship between PCs and HCs. Educational level data was missing for two patients. FBG levels were missing for one participant each in the PC and HC groups, respectively. Abbreviations: PC, pancreatic cancer; HC, healthy control; OR, odds ratio; CI, confidence interval; Ref, reference value; BMI, body mass index; FBG, fasting blood glucose; AJCC, American Joint Committee on Cancer.

**Table 2:** Unadjusted and multivariable-adjusted logistic regression analyses of FBG for PC risk.

Category		Logistic Regression	Group		Univariable		Multivariable	
			PC	HC	OR (95% CI)	*p*	OR (95% CI)	*p* ^a^
FBG	All	Continuous scale	54	279	1.03 (1.02~1.04)	<0.0001	1.04 (1.03~1.05)	<0.0001
		FBG (≥126 mg/dL)	28	26	10.5 (5.37~20.5)	<0.0001	13.4 (6.24~28.7)	<0.0001
		FBG (<126 mg/dL)	26	253	Ref		Ref	
	Male	Continuous scale	26	134	1.03 (1.01~1.04)	<0.0001	1.03 (1.02~1.05)	<0.0001
		FBG (≥126 mg/dL)	14	20	6.65 (2.69~16.4)	<0.0001	7.70 (2.65~22.5)	0.0002
		FBG (<126 mg/dL)	12	114	Ref		Ref	
	Female	Continuous scale	28	145	1.05 (1.02~1.07)	<0.0001	1.05 (1.03~1.08)	<0.0001
		FBG (≥126 mg/dL)	14	6	23.2 (7.69~69.8)	<0.0001	32.3 (9.28~112.4)	<0.0001
		FBG (<126 mg/dL)	14	139	Ref		Ref	

ORs and 95% CIs were evaluated for analyzing the relationship between FBG concentration and PC risk using univariate and multivariate logistic regression analyses. FBG levels were categorized as ≥126 mg/dL and <126 mg/dL. ^a^ *p*-Values were adjusted for sex, age, BMI, and smoking status. One FBG data value was missing from each of the two groups. Abbreviations: FBG, fasting blood glucose; PC, pancreatic cancer; HC, healthy control; OR, odds ratio; CI, confidence interval; Ref, reference value.

**Table 3:** Unadjusted and multivariable-adjusted logistic regression analyses of the combined exposures for PC risk.

Exposure		Group		Univariable		Multivariable	
		PC	HC	OR (95% CI)	*p*	OR (95% CI)	*p* ^a^
Low DII	with FBG <126 mg/dL	10	134	1 (Ref)		1 (Ref)	
	with FBG ≥126 mg/dL	7	16	5.86 (1.96~17.6)	0.001	7.50 (2.31~24.4)	0.0008
High DII	with FBG <126 mg/dL	16	119	1.80 (0.79~4.12)	0.16	1.76 (0.64~4.80)	0.27
	with FBG ≥126 mg/dL	21	10	28.1 (10.5~75.7)	<0.0001	32.5 (9.85~106.9)	<0.0001
*p* for trend					<0.0001		<0.0001
RERI/S					21.5/4.79		24.2/4.34
*p* for interaction					0.01		0.04
Low DII	with non-smoker	8	82	1 (Ref)		1 (Ref)	0.39
	with ex/current smoker	6	69	1.34 (0.49~3.65)	0.57	1.67 (0.51~5.46)	0.13
High DII	with non-smoker	18	76	2.43 (1.00~2.77)	0.05	2.20 (0.79~6.16)	0.02
	with ex/current smoker	20	53	3.87 (1.59~9.42)	0.002	3.88 (1.25~12.0)	0.01
*p* for trend					0.0009		0.01
RERI/S					1.10/1.63		1.01/1.54
*p* for interaction					0.58		0.76

ORs and 95% CIs were evaluated to examine the relationship between the DII score and FBG levels or smoking status for PC risk using a multivariate logistic regression analysis. The DII scores were divided into two ranges based on the median value: high and low. ^a^ *p*-Values were adjusted for sex, age, BMI, smoking status, and energy intake. The *p*-values for trends were computed by treating the values as continuous variables, evaluating the continuous scale, and allocating median values to every quantile. The combined effect was assessed through additive interactions using RERI/S and the *p*-values for multiplicative interactions from dichotomized exposures. Values of RERI > 0 and S > 1 indicate a synergistic interaction between the two factors. Abbreviations: PC, pancreatic cancer; HC, healthy control; OR, odds ratio; CI, confidence interval; DII, dietary inflammatory index; FBG, fasting blood glucose; Ref, reference value; RERI, relative excess risk due to interaction; S, synergy index.

## Data Availability

The data presented in this study are available upon formal request from the corresponding author.

## Supplementary Materials

The following supporting information can be downloaded at: https://www.mdpi.com/article/10.3390/nu16223941/s1, Table S1: Unadjusted and multivariable-adjusted logistic regression analysis of three levels of E-DII score for PC risk by sex; Table S2: Unadjusted and multivariable-adjusted logistic regression analysis of 30 food parameters of DII for PC by tertile; Figure S1: Adjusted Cox proportional hazards model for 5-year DFS, 5-year OS, and 5-year RFS of patients with PC by FBG; Figure S2: Adjusted Cox proportional hazards model for 5-year DFS, 5-year OS, and 5-year RFS of patients with PC by smoking status; Figure S3: The hypothesis of the underlying mechanism for the synergistic effect of high FBG and high DII on the risk of PC.
