# Supplementary material for: The Impact of the Dietary Inflammatory Index, Fasting Blood Glucose, and Smoking Status on the Incidence and Survival of Pancreatic Cancer: A Retrospective Case–Control Study and a Prospective Study
Source: Nutrients. 2024 Nov 19;16(22):3941. doi: 10.3390/nu16223941 (PMC11597200; doi:10.3390/nu16223941)
Supplement: Supplementary file 1 [file nutrients-16-03941-s001.zip › nutrients-3279452-supplementary.pdf]

Supplementary Table S1. Unadjusted and multivariable-adjusted logistic regression analysis of three-levels of E-DII score for PC risk by sex.

Category		Logistic regression	Group		Univariable			Multivariable		
			**PC**	**HC**	**OR (95% CI)**	***p***	***p* for trend**	**OR (95%CI)**	***p* [a]**	***p* for trend**
E-DII	All	Continuous scale	55	280	1.32 (1.13~1.54)	0.0004	0.0003	1.28 (1.08~1.51)	0.01	0.002
		Tertile3(>-1.24)	31	94	2.79 (1.32~5.87)	0.007		2.41 (1.10~5.30)	0.10	
		Tertile2(-2.59--1.24)	13	93	1.18 (0.50~2.77)	0.70	0.0006	1.13 (0.47~2.70)	0.83	0.003
		Tertile1(<-2.59)	11	93	Ref			Ref		
	Male	Continuous scale	27	135	1.23 (1.00~1.51)	0.05	0.05	1.16 (0.92~1.45)	0.35	0.23
		Tertile3(>-1.04)	15	45	3.75 (1.15~12.2)	0.03		2.88 (0.84~9.86)	0.28	
		Tertile2(-2.56--1.04)	8	45	2.00 (0.56~7.12)	0.28	0.18	2.06 (0.55~7.73)	0.43	0.53
		Tertile1(<-2.56)	4	45	Ref			Ref		
	Female	Continuous scale	28	145	1.46 (1.15~1.85)	0.002	0.001	1.46 (1.13~1.90)	0.02	0.002
		Tertile3(>-1.40)	17	49	2.38 (0.91~6.25)	0.08		2.37 (0.85~6.63)	0.30	
		Tertile2(-2.60--1.40)	4	48	0.57 (0.16~2.08)	0.40	0.002	0.54 (0.15~1.99)	0.53	0.002
		Tertile1(<-2.60)	7	48	Ref			Ref		

ORs and 95% CIs were evaluated the relationship between E-DII score and PC risk using univariable and multivariable logistic regression analysis. E-DII expressed as tertiles. Tertile 1 represents the lowest E-DII, the most anti-inflammatory diet, while tertile 3 represents the highest E-DII, the most pro-inflammatory diet. [a] *p*-Values were adjusted for gender, age, BMI, and smoking. *p* for trends was computed by treating the values as continuous variables, evaluating the continuous scale, and allocating median values to every quantile. Abbreviations: OR, odds ratio; CI, confidence interval; E-DII, energy-adjusted dietary inflammatory index; PC, pancreatic cancer; HC, healthy control; Ref, reference value.

Supplement Table S2. Unadjusted and multivariable-adjusted logistic regression analysis of 30-food parameters of DII for PC by tertile.

Category	Logistic regression	Group		Unadjusted		Multivariable Adjusted	
		PC	HC	OR (95% CI)	*p*	OR (95% CI)	*p* [a]
Energy (kcal)	Continuous scale	55	280	1.00 (1.00~1.00)	0.06	1.00 (1.00~1.00)	0.28
	Tertile3(>2434.58)	14	94	0.58 (0.28~1.18)	0.13	0.71 (0.33~1.53)	0.53
	Tertile2(1816.97-2434.58)	17	93	0.71 (0.36~1.40)	0.32	0.80 (0.38~1.68)	0.65
	Tertile1(<1816.97)	24	93	Ref		Ref	
Carbohydrates (g)	Continuous scale	55	280	1.00 (1.00~1.00)	0.07	1.00 (1.00~1.00)	0.24
	Tertile3(>386.83)	13	94	0.48 (0.23~0.98)	0.04	0.53 (0.25~1.12)	0.14
	Tertile2(283.61-386.83)	15	93	0.56 (0.28~1.11)	1.00	0.57 (0.27~1.18)	0.15
	Tertile1(<283.61)	27	93	Ref		Ref	
Total fat (g)	Continuous scale	55	280	0.98 (0.97~1.00)	0.02	0.99 (0.97~1.00)	0.09
	Tertile3(>53.38)	12	94	0.42 (0.20~0.88)	0.02	0.51 (0.24~1.10)	0.12
	Tertile2(35.82-53.38)	15	93	0.54 (0.27~1.07)	0.08	0.53 (0.25~1.09)	0.10
	Tertile1(<35.82)	28	93	Ref		Ref	
Protein (g)	Continuous scale	55	280	1.00 (0.99~1.01)	0.48	1.00 (0.99~1.01)	0.95
	Tertile3(>85.35)	18	94	0.81 (0.41~1.61)	0.55	1.01 (0.49~2.10)	0.98
	Tertile2(60.56-85.35)	15	93	0.68 (0.33~1.40)	0.29	0.72 (0.34~1.55)	0.57
	Tertile1(<60.56)	22	93	Ref		Ref	
Fiber (g)	Continuous scale	55	280	0.99 (0.96~1.01)	0.18	0.99 (0.97~1.02)	0.62
	Tertile3(>35.32)	12	94	0.49 (0.23~1.05)	0.07	0.58 (0.26~1.28)	0.24
	Tertile2(23.69-35.32)	19	93	0.79 (0.41~1.54)	0.49	0.90 (0.45~1.82)	0.87
	Tertile1(<23.69)	24	93	Ref		Ref	
Vitamin A (RE)	Continuous scale	55	280	1.00 (1.00~1.00)	<0.0001	1.00 (1.00~1.00)	0.0006
	Tertile3(>1429.73)	7	94	0.17 (0.07~0.41)	<0.0001	0.19 (0.08~0.47)	0.001
	Tertile2(871.95-1429.73)	8	93	0.20 (0.09~0.45)	0.0001	0.21 (0.09~0.49)	0.002
	Tertile1(<871.95)	40	93	Ref		Ref	
β-carotene	Continuous scale	55	280	1.00 (1.00~1.00)	<0.0001	1.00 (1.00~1.00)	0.0003

(μg)	Tertile3(>8054.15)	6	94	0.14 (0.06~0.34)	<0.0001	0.16 (0.06~0.41)	0.001
	Tertile2(4753.30-8054.15)	8	93	0.20 (0.09~0.44)	<0.0001	0.21 (0.09~0.49)	0.001
	Tertile1(<4753.30)	41	93	Ref		Ref	
Vitamin D (μg)	Continuous scale	55	280	0.51 (0.40~0.65)	<0.0001	0.51 (0.39~0.65)	<0.0001
	Half2(>3.63)	3	140	0.06 (0.02~0.19)	<0.0001	0.05 (0.02~0.18)	<0.0001
	Half1(≤3.63)	52	140	Ref		Ref	
Vitamin E (mg)	Continuous scale	55	280	0.91 (0.87~0.95)	<0.0001	0.91 (0.87~0.95)	0.0002
	Tertile3(>24.94)	5	94	0.12 (0.05~0.33)	<0.0001	0.14 (0.05~0.39)	0.0009
	Tertile2(16.86-24.94)	10	93	0.25 (0.12~0.53)	0.0003	0.28 (0.13~0.60)	0.005
	Tertile1(<16.86)	40	93	Ref		Ref	
Vitamin C (mg)	Continuous scale	55	280	0.99 (0.98~0.99)	<0.0001	0.99 (0.98~0.99)	<0.0001
	Half2(>144.33)	9	140	0.20 (0.09~0.41)	<0.0001	0.18 (0.08~0.40)	0.0002
	Half1(≤144.33)	46	140	Ref		Ref	
Thiamin (mg)	Continuous scale	55	280	0.18 (0.10~0.32)	<0.0001	0.20 (0.11~0.36)	<0.0001
	Half2(>1.92)	7	140	0.15 (0.06~0.33)	<0.0001	0.16 (0.07~0.37)	0.0001
	Half1(≤1.92)	48	140	Ref		Ref	
Riboflavin (mg)	Continuous scale	55	280	1.11 (0.75~1.64)	0.62	1.33 (0.87~2.02)	0.22
	Tertile3(>1.84)	19	94	0.99 (0.49~1.99)	0.98	1.22 (0.58~2.54)	0.84
	Tertile2(1.28-1.84)	17	93	0.89 (0.44~1.83)	0.76	0.92 (0.43~1.95)	0.91
	Tertile1(<1.28)	19	93	Ref		Ref	
Niacin (mg)	Continuous scale	55	280	0.90 (0.85~0.95)	<0.0001	0.91 (0.86~0.96)	0.006
	Tertile3(>20.59)	9	94	0.27 (0.12~0.60)	0.001	0.33 (0.15~0.76)	0.02
	Tertile2(14.39-20.59)	13	93	0.39 (0.20~0.80)	0.009	0.41 (0.19~0.86)	0.03
	Tertile1(<14.39)	33	93	Ref		Ref	
Vitamin B6 (mg)	Continuous scale	55	280	0.0003 (0.00~0.004)	<0.0001	0.0003 (0.00~0.005)	<0.0001
Folic acid (μg)	Continuous scale	55	280	0.99 (0.99~0.99)	<0.0001	0.99 (0.99~0.99)	<0.0001
	Half2(>623.98)	5	140	0.10 (0.04~0.26)	<0.0001	0.10 (0.04~0.27)	<0.0001
	Half1(≤623.98)	50	140	Ref		Ref	

Vitamin B12 (μg)	Continuous scale	55	280	0.53 (0.44~0.64)	<0.0001	0.52 (0.43~0.63)	<0.0001
Magnesium (mg)	Continuous scale	55	280	1.01 (1.01~1.02)	<0.0001	1.02 (1.01~1.02)	<0.0001
	Half2(>143.46)	49	140	8.17 (3.39~19.7)	<0.0001	8.36 (3.38~20.7)	<0.0001
	Half1(≤143.46)	6	140	Ref		Ref	
Iron (mg)	Continuous scale	55	280	0.86 (0.87~0.91)	<0.0001	0.86 (0.81~0.92)	<0.0001
	Tertile3(>20.48)	5	94	0.12 (0.04~0.31)	<0.0001	0.12 (0.05~0.34)	0.0003
	Tertile2(13.61-20.48)	8	93	0.19 (0.08~0.43)	<0.0001	0.22 (0.09~0.50)	0.001
	Tertile1(<13.61)	42	93	Ref		Ref	
Zinc (mg)	Continuous scale	55	280	0.92 (0.86~0.99)	0.02	0.94 (0.87~1.01)	0.08
	Tertile3(>13.96)	11	94	0.38 (0.18~0.80)	0.01	0.45 (0.21~0.99)	0.08
	Tertile2(9.90-13.96)	15	93	0.52 (0.26~1.03)	0.06	0.61 (0.30~1.27)	0.22
	Tertile1(<9.90)	29	93	Ref		Ref	
Selenium (μg)	Continuous scale	55	280	1.00 (0.99~1.01)	0.76	1.00 (0.99~1.01)	0.94
	Tertile3(>103.22)	18	94	0.89 (0.44~1.80)	0.74	1.05 (0.50~2.21)	0.95
	Tertile2(74.24-103.22)	17	93	0.85 (0.42~1.72)	0.65	0.95 (0.45~2.02)	0.95
	Tertile1(<74.24)	20	93	Ref		Ref	
Cholesterol (mg)	Continuous scale	55	280	0.99 (0.99~0.99)	<0.0001	0.99 (0.99~1.00)	<0.0001
	Half2(>291.03)	9	140	0.20 (0.09~0.41)	<0.0001	0.20 (0.09~0.44)	0.0003
	Half1(≤291.03)	46	140	Ref		Ref	
SFA (g)	Continuous scale	55	280	0.95 (0.90~1.00)	0.04	0.96 (0.91~1.01)	0.15
	Tertile3(>14.09)	12	94	0.47 (0.23~1.00)	0.05	0.53 (0.24~1.16)	0.16
	Tertile2(8.95-14.09)	18	93	0.72 (0.37~1.41)	0.34	0.80 (0.40~1.63)	0.64
	Tertile1(<8.95)	25	93	Ref		Ref	
MUFA (g)	Continuous scale	55	280	0.88 (0.83~0.93)	<0.0001	0.89 (0.84~0.94)	0.0003
	Half2(>13.81)	12	140	0.28 (0.14~0.55)	0.0002	0.31 (0.15~0.63)	0.004
	Half1(≤13.81)	43	140	Ref		Ref	
PUFA (g)	Continuous scale	55	280	0.95 (0.91~0.99)	0.02	0.96 (0.92~1.00)	0.08
	Tertile3(>17.17)	10	94	0.45 (0.20~1.00)	0.05	0.52 (0.22~1.19)	0.17

	Tertile2(10.66-17.17)	23	93	1.05 (0.55~2.01)	0.89	1.16 (0.58~2.32)	0.79
	Tertile1(<10.66)	22	93	Ref		Ref	
n-3 PUFA (g)	Continuous scale	55	280	2.21 (1.60~3.05)	<0.0001	2.44 (1.72~3.48)	<0.0001
	Half2(>0.90)	46	140	5.11 (2.41~10.8)	<0.0001	6.00 (2.70~13.4)	<0.0001
	Half1(≤0.90)	9	140	Ref		Ref	
n-6 PUFA (g)	Continuous scale	55	280	1.23 (1.15~1.30)	<0.0001	1.26 (1.17~1.35)	<0.0001
	Half2(>4.02)	51	140	12.8 (4.49~36.2)	<0.0001	13.0 (4.47~38.1)	<0.0001
	Half1(≤4.02)	4	140	Ref		Ref	
Garlic (g)	Continuous scale	55	280	1.00 (0.97~1.03)	0.95	1.01 (0.98~1.05)	0.59
	Tertile3(>2.52)	24	79	3.01 (1.36~6.66)	0.01	3.87 (1.66~9.00)	0.004
	Tertile2(0.42-2.52)	21	102	2.04 (0.91~4.55)	0.08	2.65 (1.13~6.22)	0.03
	Tertile1(<0.42)	10	99	Ref		Ref	
Tea (g)	Continuous scale	55	280	0.98 (0.97~1.00)	0.07	0.98 (0.91~1.00)	0.04
	Tertile3(>12.83)	12	79	0.67 (0.31~1.44)	0.31	0.60 (0.27~1.34)	0.30
	Tertile2(0.85-12.83)	21	104	0.89 (0.41~1.72)	0.73	0.95 (0.42~1.93)	0.89
	Tertile1(<0.85)	22	97	Ref		Ref	
Caffeine (g)	Continuous scale	55	280	0.05 (0.00~34.5)	0.37	0.11 (0.00~96.9)	0.62
	Tertile3(>0.06)	14	91	0.59 (0.29~1.20)	0.15	0.66 (0.31~1.43)	0.41
	Tertile2(0.03-0.06)	15	89	0.65 (0.32~1.30)	0.22	0.73 (0.35~1.50)	0.45
	Tertile1(<0.03)	26	100	Ref		Ref	
Alcohol (g)	Continuous scale	55	280	1.00 (1.00~1.00)	0.82	1.00 (1.00~1.00)	0.36
	Tertile3(>70.42)	16	94	0.58 (0.30~1.14)	0.12	0.94 (0.30~2.98)	0.96
	Tertile2(0.00-70.42)	9	83	0.37 (0.17~0.83)	0.02	0.73 (0.22~2.46)	0.86
	Tertile1(<0.00)	30	103	Ref		Ref	

ORs and 95% CI were used to evaluate the relationship between DII score and PC risk using univariable and multivariable logistic regression analysis. The data was stratified into tertiles (tertile1, tertile2, and tertile3) or half (half1 and half2). [a] *p*-Values were adjusted for gender, age, BMI, smoking, and energy intake. Abbreviations: OR, odds ratio; CI, confidence interval; DII, dietary inflammatory index; PC, pancreatic cancer; HC, healthy control; SFA, saturated fatty acid; MUFA, monounsaturated fatty acids; PUFA, polyunsaturated fatty acid; Ref, reference value.

Supplementary Figure S1. Adjusted Cox proportional-hazards model for 5-year DFS, 5-year OS, and 5-year RFS of patients with PC by FBG.

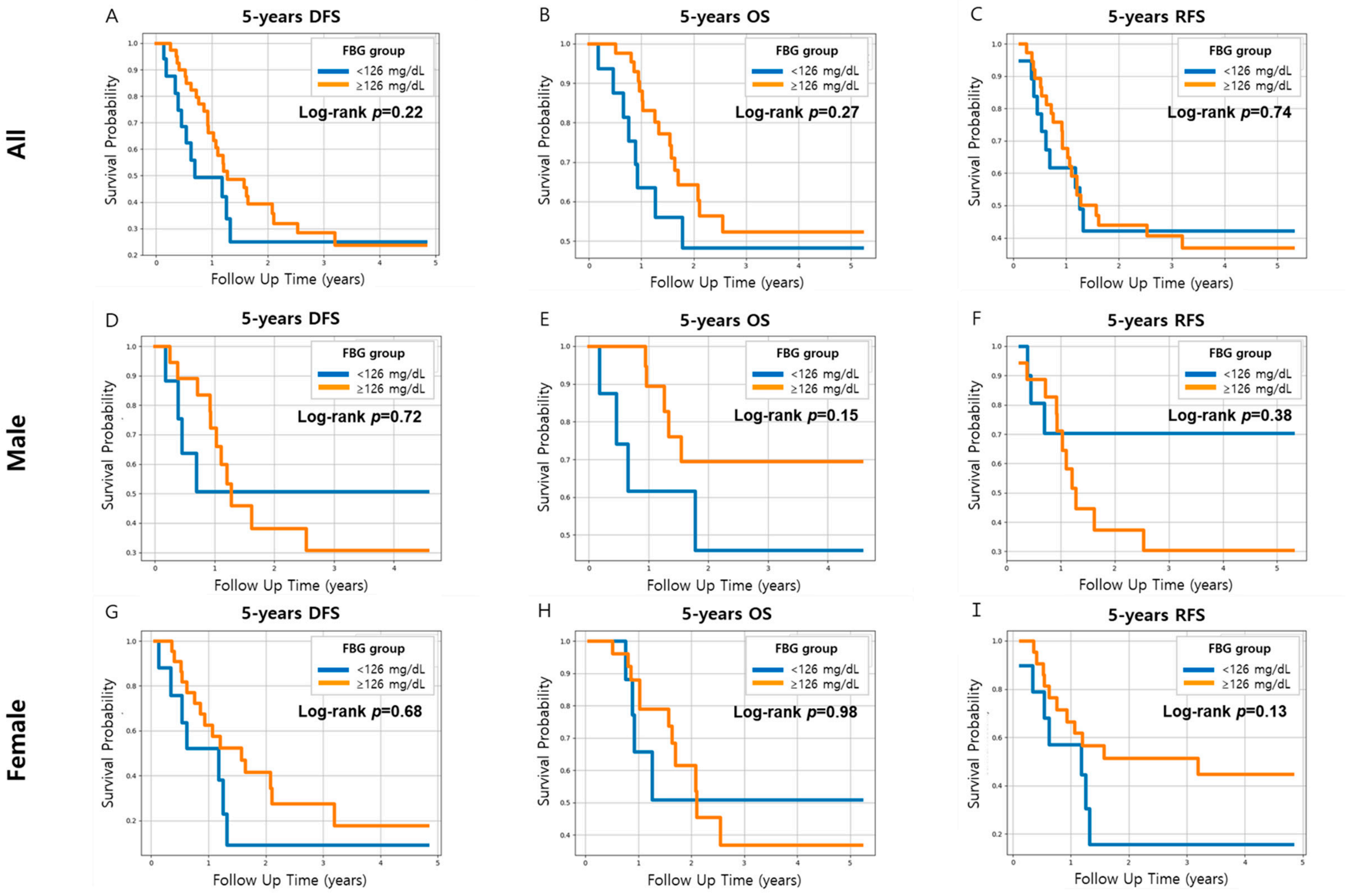

The prognosis of the 55 PC cases was analyzed to assess DFS, OS, and RFS using the Cox proportional hazards model. FBG was divided into 2 groups: FBG < 126 mg/dL and FBG ≥ 126 mg/dL. Survival curves based on FBG level was analyzed among 55 PCs for (a) 5-years DFS, (b) 5-years OS, (c) 5-years RFS, (d) 5-years DFS for male, (e) 5-years OS for male, and (f) 5-years RFS for male, (g) 5-years DFS for female, (h) 5-years OS for female, and (i) 5-years RFS for female. Abbreviations: DFS, disease-free survival; OS, overall survival; RFS, recurrence-free survival; PC, pancreatic cancer; FBG, fasting blood glucose.

Supplementary Figure S2. Adjusted Cox proportional-hazards model for 5-year DFS, 5-year OS, and 5-year RFS of patients with PC by smoking status.

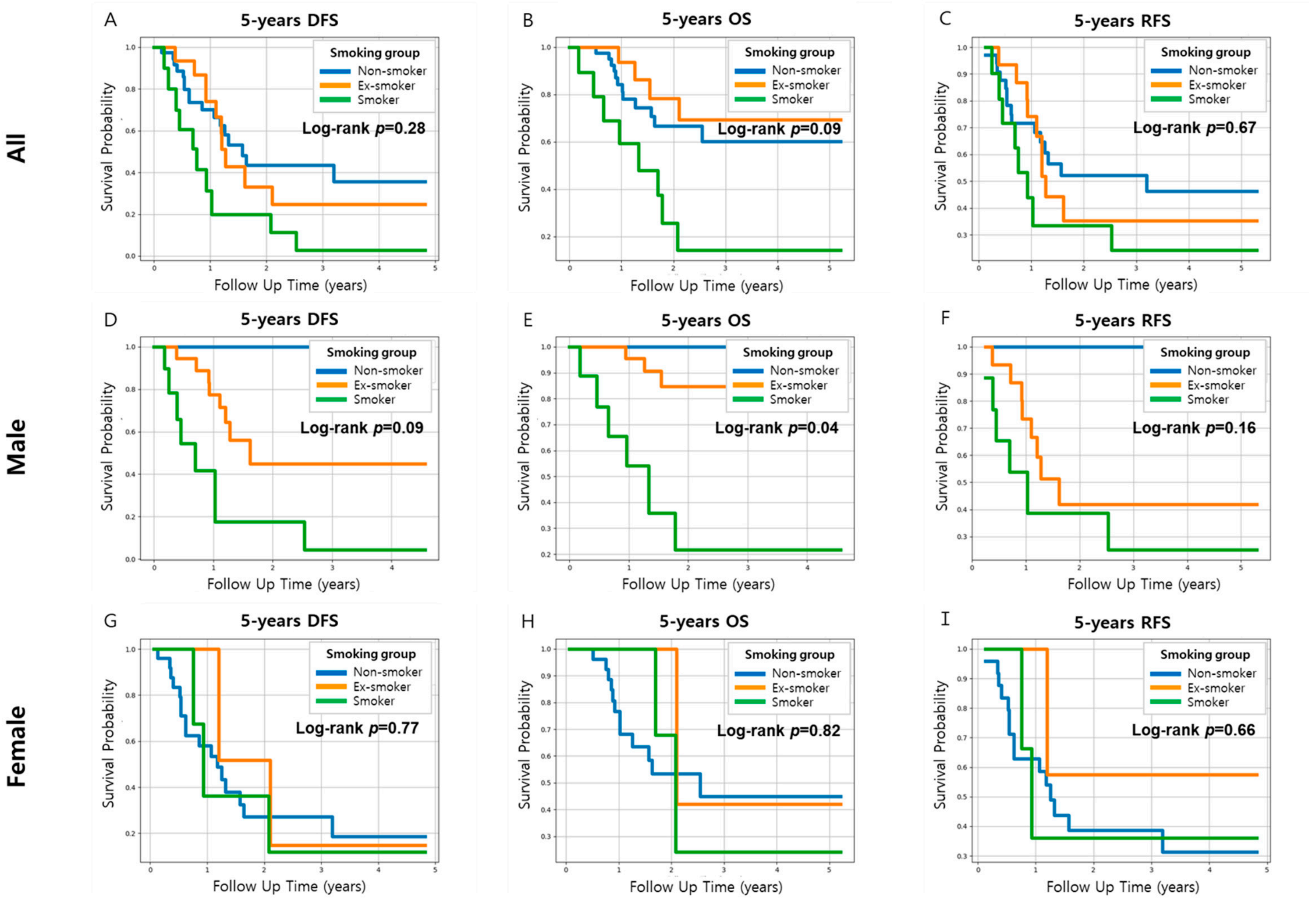

The prognosis of the 55 PC cases was analyzed to assess DFS, OS, and RFS using the Cox proportional-hazards model. Smoking status was divided into 3 groups: no-smoker, ex-smoker, and smoker. Survival curves based on smoking status was analyzed among 55 PCs for (a) 5-years DFS, (b) 5-years OS, (c) 5-years RFS, (d) 5-years DFS for male, (e) 5-years OS for male, and (f) 5-years RFS for male, (g) 5-years DFS for female, (h) 5-years OS for female, and (i) 5-years RFS for female. Abbreviations: DFS, disease-free survival; OS, overall survival; RFS, recurrence-free survival; PC, pancreatic cancer.

Supplementary Figure S3. Hypothesis of the underlying mechanism for the synergistic effect of high-FBG and high-DII on the risk of PC.

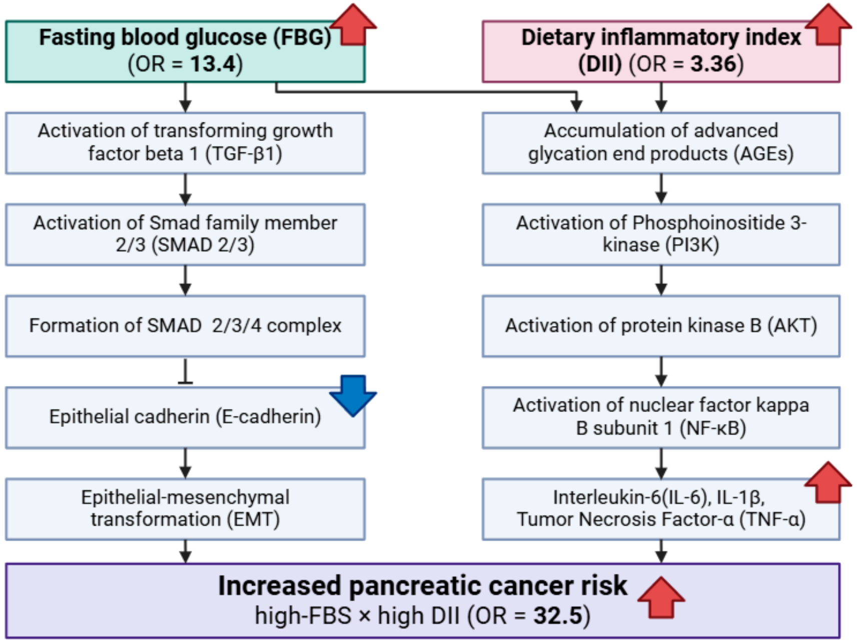

Abbreviations: FBG, fasting blood glucose; DII, dietary inflammatory index; PC, pancreatic cancer; OR, odds ratio.
